# Genetic context determines susceptibility to intraocular pressure elevation in a mouse pigmentary glaucoma

**DOI:** 10.1186/1741-7007-4-20

**Published:** 2006-07-07

**Authors:** Michael G Anderson, Richard T Libby, Mao Mao, Ioan M Cosma, Larry A Wilson, Richard S Smith, Simon WM John

**Affiliations:** 1 The Jackson Laboratory, Bar Harbor, ME, USA; 2 Department of Physiology and Biophysics, University of Iowa, Iowa City, IA, USA; 3 Howard Hughes Medical Institute, Bar Harbor, ME, USA; 4 Tufts University School of Medicine, Boston, MA, USA

## Abstract

**Background:**

DBA/2J (D2) mice develop an age-related form of glaucoma. Their eyes progressively develop iris pigment dispersion and iris atrophy followed by increased intraocular pressure (IOP) and glaucomatous optic nerve damage. Mutant alleles of the *Gpnmb *and *Tyrp1 *genes are necessary for the iris disease, but it is unknown whether alleles of other D2 gene(s) are necessary for the distinct later stages of disease. We initiated a study of congenic strains to further define the genetic requirements and disease mechanisms of the D2 glaucoma.

**Results:**

To further understand D2 glaucoma, we created congenic strains of mice on the C57BL/6J (B6) genetic background. B6 double-congenic mice carrying D2-derived *Gpnmb *and *Tyrp1 *mutations develop a D2-like iris disease. B6 single-congenics with only the *Gpnmb *and *Tyrp1 *mutations develop milder forms of iris disease. Genetic epistasis experiments introducing a B6 tyrosinase mutation into the congenic strains demonstrated that both the single and double-congenic iris diseases are rescued by interruption of melanin synthesis. Importantly, our experiments analyzing mice at ages up to 27 months indicate that the B6 double-congenic mice are much less prone to IOP elevation and glaucoma than are D2 mice.

**Conclusion:**

As demonstrated here, the *Gpnmb *and *Tyrp*1 iris phenotypes are both individually dependent on tyrosinase function. These results support involvement of abnormal melanosomal events in the diseases caused by each gene. In the context of the inbred D2 mouse strain, the glaucoma phenotype is clearly influenced by more genes than just *Gpnmb *and *Tyrp1*. Despite the outward similarity of pigment-dispersing iris disease between D2 and the B6 double-congenic mice, the congenic mice are much less susceptible to developing high IOP and glaucoma. These new congenic strains provide a valuable new resource for further studying the genetic and mechanistic complexity of this form of glaucoma.

## Background

The glaucomas are a common group of diseases sharing a characteristic pattern of retinal ganglion cell (RGC) degeneration and optic nerve head excavation [1-3]. Increased intraocular pressure (IOP) is a significant predisposing risk factor for glaucoma, and all current glaucoma treatments aim to manipulate this trait [4,5]. Pigment dispersion syndrome (PDS) is a common condition that results in the dispersion of iris pigment into the anterior chamber (AC) [6,7]. The dispersed pigment accumulates within the ocular drainage structures, resulting in IOP elevation and glaucoma in some but not all individuals with PDS [8-12]. The molecular events that underlie PDS and that determine whether pigment dispersion leads to IOP elevation and pigmentary glaucoma remain largely unknown. A promising approach for gaining insight into these complex processes is to use the mouse as an experimental system [13-16]. A benefit of this approach is that genetic and environmental factors can be significantly controlled and manipulated, thus making the biological complexity of a disease such as glaucoma easier to approach experimentally.

DBA/2J (D2) mice, and some related substrains, develop a form of glaucoma characterized by a pigment-dispersing iris disease that aberrantly deposits pigment throughout the AC, including the drainage structures of the eye. As a consequence, IOP becomes elevated and glaucomatous RGC degeneration ensues [17-21]. The D2 iris disease is initiated by the combined interactions of the *Tyrp1*^*b *^and *Gpnmb*^*R150X *^mutations [22,23]. *Tyrp1 *encodes a membrane-bound melanosomal protein with enzymatic activity involved in melanin synthesis [24-26]. Relatively little is known about *Gpnmb *function. The GPNMB protein has been detected in melanocytes [27], dendritic cells [28], and a small list of other cell types [29-33]. Despite these findings, the cellular mechanisms by which *Tyrp1 *and *Gpnmb *contribute to the glaucoma remain largely unknown. Furthermore, it is also unknown whether the *Gpnmb *and *Tyrp1 *mutations are sufficient to elicit the D2 form of glaucoma, or whether additional alleles are necessary during distinct stages of disease.

Here, we report experiments utilizing an analysis of congenic strains of mice that further probe the genetic requirements contributing to the D2 form of glaucoma. The D2-derived *Tyrp1*^*b *^and *Gpnmb*^*R150X *^mutations were backcrossed into the widely used C57BL/6J (B6) background. B6 double-congenic mice (homozygous for both mutations) were utilized in genetic epistasis experiments and compared with D2 with respect to clinical indices of iris disease, IOP elevation, and optic nerve damage. Similar to D2 mice, B6 double-congenic mice develop a severe depigmenting iris disease leading to massive pigment dispersion and aberrant pigment deposition on AC tissues. In comparison to D2, the B6 double-congenic mice are much less likely to develop increased IOP in response to this pigment dispersing disease. These results indicate that initiation (iris disease) and progression (high IOP) of glaucomatous phenotypes in D2 mice are genetically separable and that additional D2-derived hereditary factors relevant to glaucoma remain to be characterized. Identification of these factors in mice may promote understanding of factors contributing to human glaucoma and suggest improved strategies for preventing glaucoma progression.

## Results

### Generation of B6 congenic strains containing the *Tyrp1*^*b *^and *Gpnmb*^*R150X *^mutations

**Figure 1:**
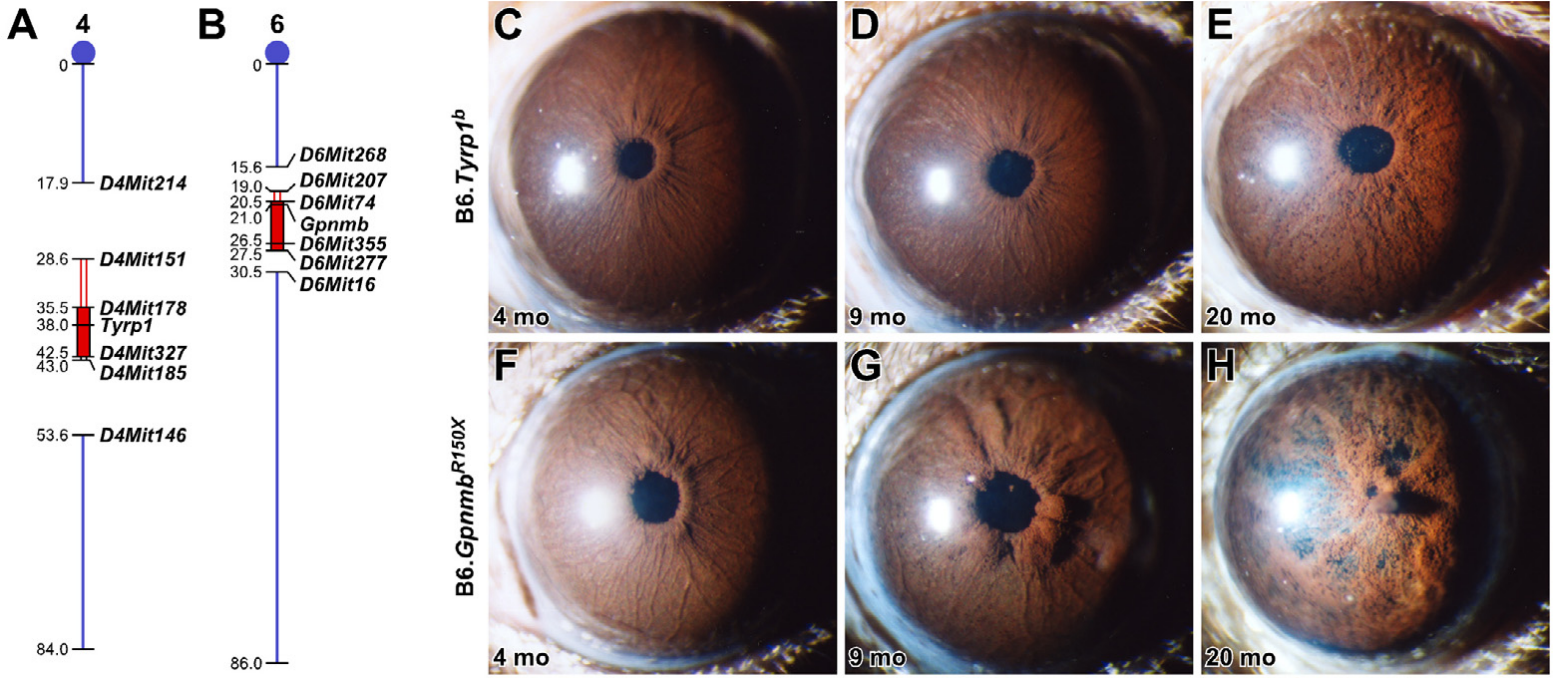
**The B6 genetic background is permissive to iris phenotypes individually caused by *Tyrp1*^*b *^and *Gpnmb*^*R150X *^mutations**. (A) The *Tyrp1*^*b *^and (B) the *Gpnmb*^*R150X *^congenic intervals on chromosome 4 and chromosome 6. After 10 generations of backcrossing, the selected D2-derived intervals (filled red box), as well as a limited amount of flanking DNA (empty red box), were present in an essentially B6-derived background (thin blue line). The chromosome 4 interval is delimited proximally by *D4Mit214*-*D4Mit151 *and distally by *D4Mit185*-*D4Mit146*; the chromosome 6 interval is delimited proximally by *D6Mit268*-*D6Mit207 *and distally by *D6Mit277*-*D6Mit16*. B6.*Tyrp1*^*b *^irides at (C) 4 months, (D) 9 months, and (E) 20 months. From 1–6 months, B6.*Tyrp1*^*b *^irides were indistinguishable from wild-type B6. Thereafter, B6.*Tyrp1*^*b *^eyes were characterized by a gradual atrophy of iris stromal morphology. Beginning at 6 months, a population of small phagocytic clump cells began to be discernible across the iris surface. With age, the underlying vasculature became obscured, and irides appeared increasingly coarse and atrophic, particularly at the pupil margin where a narrow white band of underlying tissue was exposed. With advanced age, full-thickness iris holes occurred, but rarely before 2 years of age (n = 16 eyes at 1–6 months, 20 eyes at 7–11 months, and 42 eyes at 12+ months). B6.*Gpnmb*^*R150X *^irides at (F) 4 months, (G) 9 months, and (H) 20 months. From 1–5 months, B6.*Gpnmb*^*R150X *^irides were indistinguishable from wild-type B6. Near 6 months, B6.*Gpnmb*^*R150X *^eyes developed a pronounced peripupillary swelling accompanied by pronounced accumulation of clump cells on the iris surface. These swellings remained prominent to the oldest ages examined. The iris surface maintained an overall normal morphology during the first year, after which time it became increasingly atrophic, and accumulations of dispersed pigment were visible on the lens and cornea. No sex-specific differences were evident in the phenotypes of either strain. (n = 16 eyes at 1–6 months, 20 eyes at 7–11 months, and 46 eyes at 12+ months).

In order to further probe the genetic requirements contributing to the D2 form of glaucoma, we created congenic strains of mice on the B6 genetic background. Our approach for transferring the D2-derived *Tyrp1*^*b*^and *Gpnmb*^*R150X *^mutations to the B6 background was to first create separate congenic strains for each of the respective chromosomal regions (Figure 1). Successive rounds of backcrossing utilizing markers flanking *Tyrp1 *(Figure 1A) and *Gpnmb *(Figure 1B) yielded the single-congenic strains for *Tyrp1*^*b *^(B6.D2-*Tyrp1*^*b*^/Sj; here after abbreviated B6.*Tyrp1*^*b*^) and *Gpnmb*^*R150X *^(B6.D2-*Gpnmb*^*R150X *^/Sj; here after abbreviated B6.*Gpnmb*^*R150X*^). The final sizes of the N10 congenic intervals were determined to be 14–36 cM (23.6–63.2 Mb) for B6. *Tyrp1*^*b *^and 9–15 cM (10.0–36.8 Mb) for B6. *Gpnmb*^*R150X*^. After each single N10 congenic strain was established, mice were intercrossed to generate a double-congenic stock homozygous for both congenic intervals (B6.D2-*Tyrp1*^*b*^*Gpnmb*^*R150X *^/Sj; here after abbreviated B6.*Tyrp1*^*b *^*Gpnmb*^*R150X *^or referred to as B6 double-congenic).

### B6 background is permissive to *Tyrp1*^*b *^and *Gpnmb*^*R150X *^mediated iris disease

**Figure 2:**
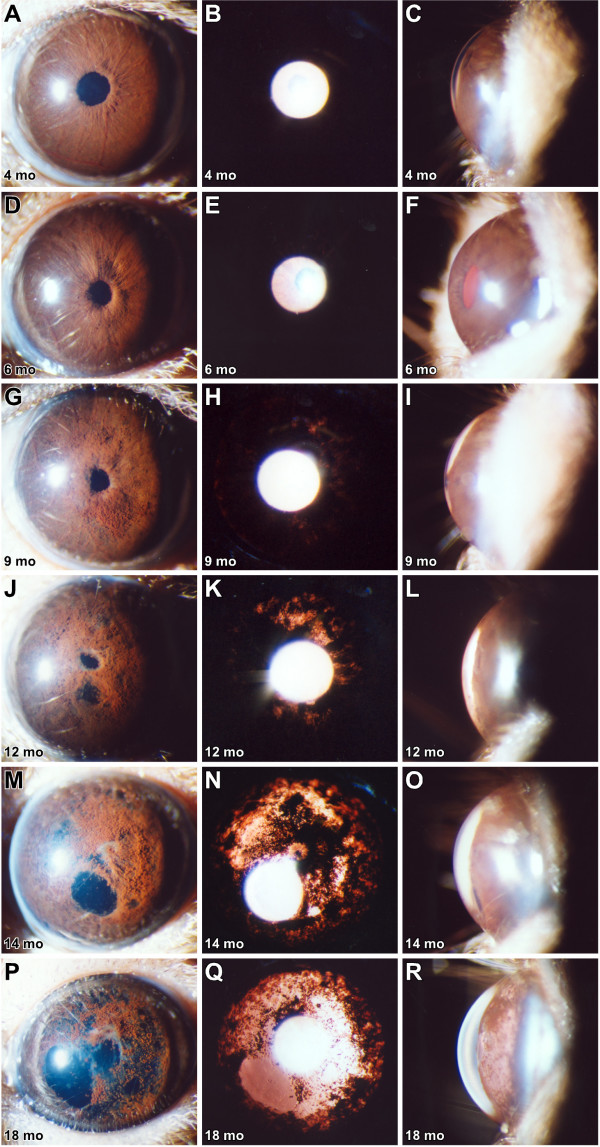
**Severe iris disease in double-congenic B6. *Tyrp1*^*b *^*Gpnmb*^*R150X *^mice**. Cohorts of B6.*Tyrp1*^*isa *^*Gpnmb*^*R150X *^mice were aged and analyzed by slit-lamp examination; representative eyes of indicated ages are shown. Each row contains three images of the same eye. The left column shows broad-beam illumination. The middle column shows transillumination defects. The right column shows the relative dimensions of the anterior chamber. The degree of pigment dispersion and iris atrophy is remarkably similar in both timing and severity to that of D2 mice (see reference [19] for comparable image of D2 eyes). (**A to C**) Until 5 months, B6.*Tyrp1*^*b*^*Gpnmb*^*R150X *^eyes were indistinguishable from wild-type, with a complex iris morphology, no transillumination, and anterior chambers of normal dimension with a closely juxtaposed cornea and iris. (**D to F**) By 6 months, all B6.*Tyrp1*^*b *^*Gpnmb*^*R150X *^eyes exhibit a clear phenotype characterized by slight swelling of peripupillary tissue. This timing and phenotype closely resembles the initial stages of the D2 iris disease. (**G to I**) In 9-month-old eyes, the peripupillary region becomes notably atrophic, transillumination is obvious, and dispersed pigment is present on both the lens and cornea. Beyond this age, a steadily worsening course ensues, which at (**J to L**) 12 months, (**M to O**) 14 months, and (**P to R**) 18 months is characterized by increasing degrees of iris atrophy that include full-thickness iris holes, profound transillumination, pigment dispersion and frequent pigment accumulation on the lens and cornea, and changes to the dimensions of the anterior chamber. No sex-specific differences were evident in these phenotypes. This synopsis of disease progression involved >146 eyes aged 2–20+ months, with each of the cohorts described above involving groups of at least 14 eyes.

Cohorts of N10 mice of all three genotypes (B6.*Tyrp1*^*b*^, B6. *Gpnmb*^*R150X*^, and B6.*Tyrp1*^*b*^*Gpnmb*^*R150X*^) were aged and assayed for relevant phenotypes. As expected, congenic mice homozygous for the *Tyrp1*^*b*^mutation had brown coat colors [34,35], whereas the *Gpnmb*^*R150X *^mutation had no effect on coat color in any of the stocks examined. Clinical analysis of iris phenotypes in aged cohorts of the single-congenic B6.*Tyrp1*^*b *^(Figs. 1C to 1E), single-congenic B6.*Gpnmb*^*R150X *^(Figs. 1F to 1H), and double-congenic B6.*Tyrp1*^*b *^*Gpnmb*^*R150X *^(Figure 2) strains of mice indicated that the B6 background was permissive to the iris disease. Iris phenotypes of the single-congenic strains closely resembled our previous observations of mice mutant for only *Gpnmb *or *Tyrp1 *in varying genetic contexts [22,23,36]. Furthermore, the timing and severity of pigment dispersing iris disease in the double-congenic B6.*Tyrp1*^*b*^*Gpnmb*^*R150X *^mice is essentially the same as in D2 mice mutant for the same *Tyrp1 *and *Gpnmb *alleles [19].

### Pigmentary pathway contributes to *Tyrp1 *and *Gpnmb *mediated iris disease

We next took advantage of the B6 background to perform strain controlled genetic epistasis experiments that tested mechanisms contributing to the *Tyrp1 *and *Gpnmb *mediated iris disease. A significant result in understanding the *Tyrp1 *and *Gpnmb *mediated iris disease is that modulating pigment production with tyrosinase (*Tyr*, a gene essential for pigment production) and hypopigmentation mutations completely rescues iris atrophy and pigment dispersion [22,23]. These experiments strongly implicated melanosomal functions of *Tyrp1 *and *Gpnmb *in pathogenesis. However, they were not absolutely complete, since only the effect of hypopigmentation mutations and not *Tyr *were assessed for *Gpnmb*. This is important because hypopigmentation mutations often affect multiple types of organelles [37,38] whereas *Tyr *has no known function in any organelles other than melanosomes.

**Figure 3:**
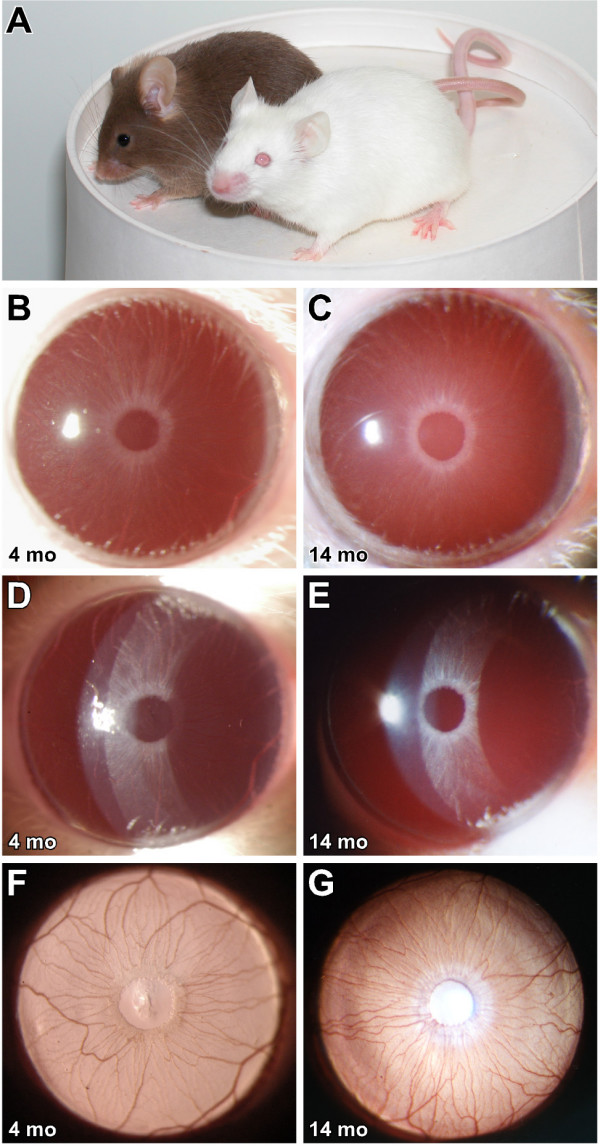
**Tyrosinase deficiency prevents iris disease in B6. *Tyrp1*^*b *^*Gpnmb*^*R150X *^mice**. Intercrosses generated mice homozygous for various genotypic combinations of the *Tyrp1*^*b *^and *Gpnmb*^*R150X *^mutations with the albino-inducing *Tyr*^*c*-2*J *^mutation. These mice were aged and analyzed by slit-lamp examination; representative B6.*Tyrp1*^*b *^*Gpnmb*^*R150X *^eyes are shown. (**A**) The normally brown pigmented coat of a mouse on the left compared with a triple homozygous *Tyr*^*c*-2*J *^*Tyrp1*^*b *^*Gpnmb*^*R150X *^mouse on the right. (**B, D, F**) Different views emphasize the clinical morphology of the albino iris. Eyes of B6 mice that are homozygous for the *Tyr*^*c*-2*J *^mutation only appear pink as they lack melanin, but otherwise the iris and its vasculature have normal morphology. (**C, E, G**) Homozygosity for *Tyr*^*c*-2*J *^completely prevents iris disease in B6.*Tyrp1*^*b *^*Gpnmb*^*R150X *^*Tyr*^*c*-2*J *^mice. The iris morphology of the triple mutant is indistinguishable from that of B6 mice homozygous for *Tyr*^*c*-2*J *^only (compare 3B to 3C), lacks peripupillary abnormalities (compare 3D to 3E), and has a healthy uninterrupted vasculature (3F to 3G). n = 14 eyes, all 12+ months.

To assess the importance of pigmentary pathways on the B6 background, we used the *Tyr*^*c*-2*J *^mutation that arose spontaneously in the B6 strain [39,40]. We generated multiple cohorts of homozygous, *Tyr*^*c*-2*J *^albino mice that were also homozygous for both *Tyrp1*^*b *^and *Gpnmb*^*R150X *^(Figure 3), or also homozygous for only *Tyrp1*^*b *^or *Gpnmb*^*R150X *^(data not shown). Aged cohorts of these albino mice were compared with normally pigmented mice of matched genotypes. All mice homozygous for the *Tyr*^*c*-2*J *^mutation had albino coats (Figure 3A) and albino irides (Figure 3B–G). Importantly, the *Tyr*^*c*-2*J *^mutation rescued all observable *Tyrp1*^*b *^– and *Gpnmb*^*R150X*^-associated iris phenotypes in double-congenic B6.*Tyrp1*^*b *^*Gpnmb*^*R150X *^mice (Figure 3C, E, and 3G) and single-congenic B6.*Tyrp1*^*b *^(n = 28 eyes, 12+ months) and B6.*Gpnmb*^*R150X *^mice (n = 8 eyes, 12+ months). These results convincingly indicate that melanin production is a necessary component of the pathological events initiating iris disease on a B6 background, and they confirm a melanosomal component for both the *Tyrp1 *and *Gpnmb *mutant phenotypes.

### B6. *Tyrp1*^*b *^and *Gpnmb*^*R150X *^mice are less susceptible to IOP elevation than D2 mice

**Figure 4:**
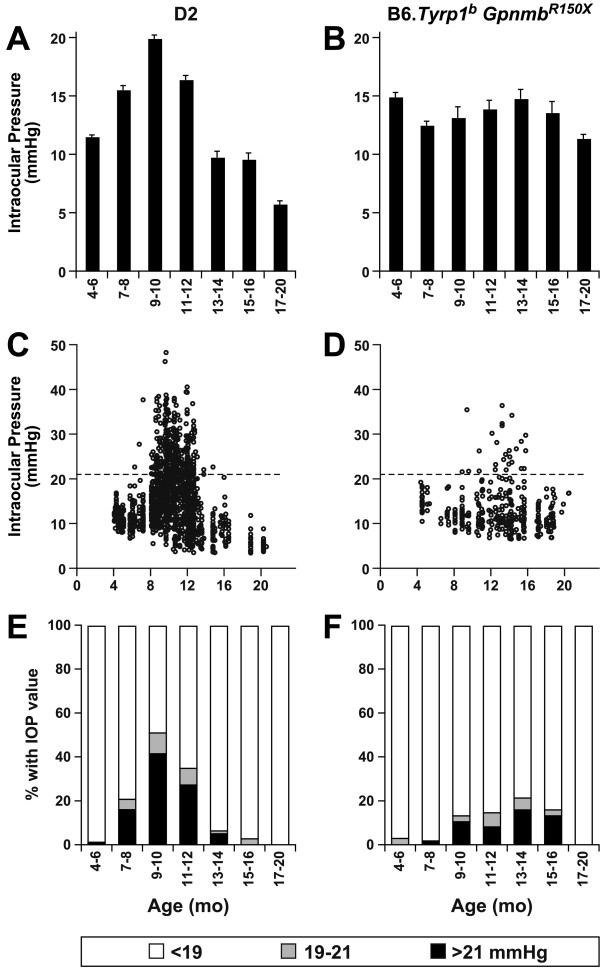
**B6.*Tyrp1*^*b *^*Gpnmb*^*R150X *^mice are relatively resistant to IOP elevation**. Graphs of IOP and age for D2 mice (left column) versus B6.*Tyrp1*^*b *^*Gpnmb*^*R150X *^mice (right column), with the same data represented as: (**A and B**) bar graphs of mean IOP +/- SEM (>30 mice per bin), (**C and D**) scatter plots, and (**E and F**) plots showing percent of mice with IOP of indicated values. IOP was recorded from a total of 336 B6.*Tyrp1*^*b *^*Gpnmb*^*R150X *^mice and 1437 D2 mice. All groups contained >30 mice and included both male and female mice.

Having established that B6.*Tyrp1*^*b*^*Gpnmb*^*R150X *^mice exhibit a pigment-dispersing iris disease that is clinically, and mechanistically, similar to that in D2 mice, we next tested whether the B6 genetic background would influence the impact of this insult on IOP. Although the timing and severity of iris disease was similar, IOP elevation was significantly muted in B6.*Tyrp1*^*b*^*Gpnmb*^*R150X *^mice compared with D2 mice (Figure 4). We compared the IOP distributions of these strains in relation to IOP values of 19 mmHg and 21 mmHg. As a strain, young D2 mice have lower IOP than young B6 mice and 19 mmHg is > 2 SD above the mean of young pre-glaucomatous D2 mice. The value of 21 mmHg is glaucoma relevant, as individuals with an IOP of 21 mmHg are commonly regarded as glaucoma suspect [41]. Strikingly, a far smaller proportion of B6.*Tyrp1*^*b*^*Gpnmb*^*R150X *^mice were detected with IOP > 21 mmHg compared with D2 mice (Figure 4A to 4D). For example, at 7–8 months only 2 % of B6.*Tyrp1*^*b*^*Gpnmb*^*R150X *^mice had IOP > 21 mmHg, compared with 16 % of D2 mice (P = 0.007,χ^2 ^comparing number of mice with IOP < 19 mmHg, 19–21 mmHg, and > 21 mmHg for each strain at this age). Similarly, at 9–10 months, only 11% of B6.*Tyrp1*^*b*^*Gpnmb*^*R150X *^had IOPs >21 mmHg, compared with 42 % of D2 (P < 0.0001, similar χ^2 ^comparison for 9 to 10-months mice). The same trend was observed considering all mice, irrespective of age (P < 0.0001, χ^2^). Further demonstrating their lower susceptibility to developing high IOP, pressure elevation was delayed in B6.*Tyrp1*^*b*^*Gpnmb*^*R150X *^mice compared with D2 mice (Figure 4E and 4F). Whereas the greatest proportion of D2 mice had IOP > 21 mmHg (42%) at 9–10 months, the greatest proportion of B6.*Tyrp1*^*b*^*Gpnmb*^*R150X *^mice (16%) had IOP > 21 4 months later at 13–14 months of age (P < 0.0001, χ^2 ^comparing B6.*Tyrp1*^*b *^*Gpnmb*^*R150X *^at 13–14 months with D2 at 9–10 months). Therefore, as a population, B6.*Tyrp1*^*b*^*Gpnmb*^*R150X *^mice are less susceptible to IOP elevation than D2 mice.

### B6. *Tyrp1*^*b *^and *Gpnmb*^*R150X *^mice do not develop glaucomatous nerve damage

**Figure 5:**
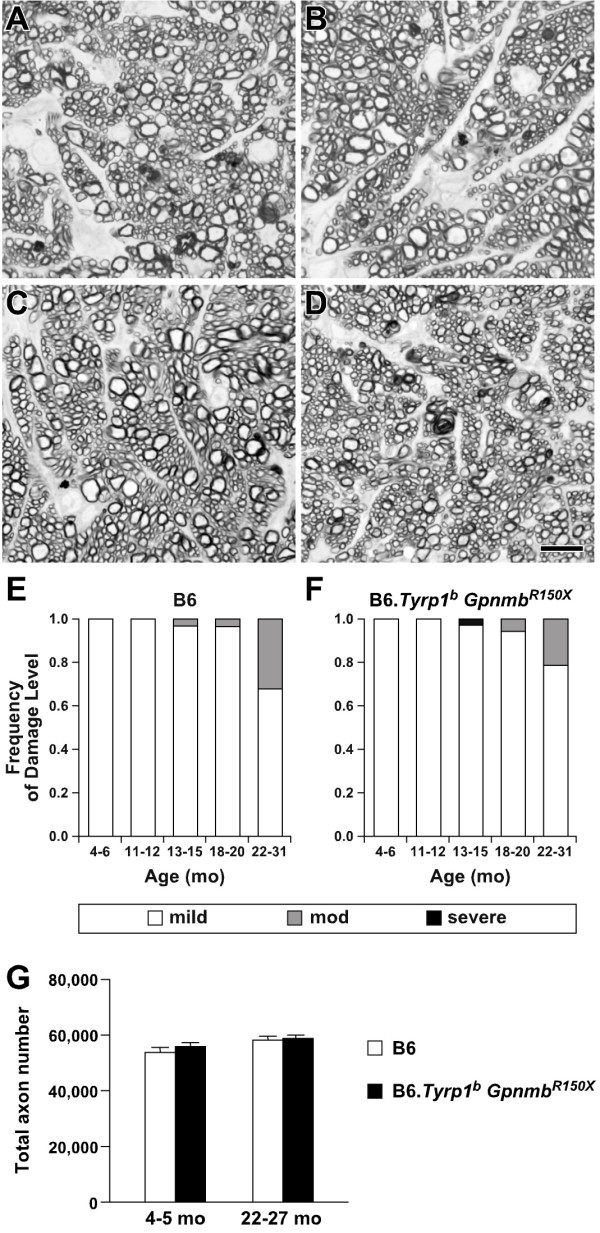
**B6. *Tyrp1*^*b *^*Gpnmb*^*R150X *^mice have optic nerve phenotypes similar to standard B6 mice**. Optic nerve cross-sections were stained by the PPD method, which darkly stains the myelin sheath of all axons and the axoplasm only of diseased or dying axons. The majority of optic nerves had only the mild degree of damage that is typical for aged mice, as shown here in a comparison of optic nerves from: (**A**) typical 22-month-old B6, versus (**B**) typical 22-month-old B6.*Tyrp1*^*b *^*Gpnmb*^*R150X*^. This was true even in mice with the highest IOPs recorded in the study, as shown here with (**C**) 14-month-old B6.*Tyrp1*^*b *^*Gpnmb*^*R150X *^and (**D**) 22-month-old B6.*Tyrp1*^*b *^*Gpnmb*^*R150X*^. Scale bar = 10 μm. (**E and F**) The overall distribution of nerve damage was almost identical in both standard B6 and B6.*Tyrp1*^*b *^*Gpnmb*^*R150X *^mice. Although some mice of each genotype developed a "moderate" degree of damage with age, the degree of damage in these mice only just met inclusion criteria for the "moderate" level and never involved significant axon loss. It was, therefore, much milder in all of these "moderate" mice than is typical of "moderate" D2 mice [42, 43]. A single B6.*Tyrp1*^*b *^*Gpnmb*^*R150X *^nerve had severe damage. The similar damage distributions of both standard B6 and B6.*Tyrp1*^*b *^*Gpnmb*^*R150X *^mice indicates that the damage reflects age-related B6 changes that are not related to *Gpnmb *and *Tyrp1 *glaucoma. For each group, the number of optic nerves graded for B6 and B6.*Tyrp1*^*b*^*Gpnmb*^*R150X *^were: (4–6 months) 10 and 8, (11–12 months) 19 and 22, (13–15 months) 29 and 38, (16–19 months) 28 and 28, (20–26 months) 24 and 17. (E) Quantitative axon counts (mean ± SEM) show no detectable axon loss in B6.*Tyrp1*^*b *^*Gpnmb*^*R150X *^compared with standard B6 mice. The similar damage distributions and lack of axon loss in both strains indicates that the detected damage reflects age-related B6 changes that are not related to *Gpnmb *and *Tyrp1 *glaucoma. For each group, the number of randomly selected nerves utilized in quantitative axon counting for B6 and B6.*Tyrp1*^*b*^*Gpnmb*^*R150X *^were: (4- months) 6 and 6, (22–27 months) 8 and 10.

The relatively low susceptibility of B6.*Tyrp1*^*b *^*Gpnmb*^*R150X *^mice to IOP elevation suggested that this strain may experience less glaucomatous nerve damage than D2 mice. In D2 mice, severe glaucomatous damage with substantial axon loss is detectable in over 50% of 11-month-old, 70% of 13-month-old and 80% of 19-month-old mice [42,43]. In contrast, severe damage was almost never detected in B6.*Tyrp1*^*b *^*Gpnmb*^*R150X *^mice (Figure 5). At ages when 50–80% of D2 mice have severe damage, only one of 109 B6.*Tyrp1*^*b*^*Gpnmb*^*R150X *^nerves was severely affected (56 of these nerves were from mice >18 months old). The distributions of nerve-damage level were almost identical in B6.*Tyrp1*^*b *^*Gpnmb*^*R150X *^and wild-type B6 mice (Figure 5), indicating that the degree of damage in B6.*Tyrp1*^*b *^*Gpnmb*^*R150X *^mice represents standard aging changes for this strain background. To maximize the likelihood of detecting glaucoma, optic nerves from the mice with the highest IOPs were examined (Figure 5C and 5D), but none had a level of damage similar to the glaucomatous damage of D2 mice. Additionally, no significant difference in the number of healthy axons was detected between normal B6 and B6.*Tyrp1*^*isa *^*Gpnmb*^*R150X *^mice aged either 4–5 or 22–27 months (Figure 5G). Combined, the analysis of optic nerves from these cohorts of B6.*Tyrp1*^*isa *^*Gpnmb*^*R150X *^mice convincingly shows that on the B6 genetic background, the *Tyrp*-*1 *and *Gpnmb*-mediated iris disease does not result in glaucoma.

## Discussion

On a D2 background, mutations in *Tyrp1 *and *Gpnmb *underlie a form of pigmentary glaucoma involving a pigment-dispersing iris disease, increased IOP, and optic-nerve disease [18,19,22,23]. Here, we report transfer of the D2-derived *Tyrp1*^*b*^and *Gpnmb*^*R150X *^mutations to the B6 genetic background to produce a B6 double-congenic strain that develops the iris disease. Surprisingly, the B6 double-congenic mice were less susceptible to IOP elevation than D2 mice and did not develop glaucomatous nerve damage. These strains provide a powerful resource for studying the functions of *Tyrp1 *and *Gpnmb*, dissecting the molecular mechanisms by which pigment dispersion leads to IOP elevation, and identifying additional genes contributing to the D2 form of glaucoma. Furthermore, congenic strains are broadly useful resources, and these strains will likely have additional benefit to the broader mouse-genetics community [44].

### Melanosomal processes contribute to both *Gpnmb *and *Tyrp1 *phenotypes

The current experiments demonstrate that melanosomal defects associated with melanin synthesis play a key role in the initiation of both *Tyrp*-*1 *and *Gpnmb*-mediated iris disease. Melanosomal synthesis of melanin involves a series of reactions creating the biopolymer melanin from the precursor tyrosine [45]. Many of the indole-quinone intermediates of these reactions are cytotoxic, and the mechanisms regulating their cytotoxicity are only partially understood [46,47]. Our results indicate that mutations in *Tyrp1 *and *Gpnmb *act in the melanosome, where they promote cytotoxicity in a manner that can be rescued by halting production of these toxic intermediates. Based on these findings, it will be important to examine other genes encoding melanosomal proteins that may participate in these events and that may also cause similar diseases of the iris.

### Genetic background affects susceptibility to IOP elevation and subsequent glaucoma

The experiments described above demonstrate that the severity and timing of the pigment-dispersing iris disease of B6 double-congenic mice is essentially the same as that of D2 mice. This suggests that the dispersed pigment and iris debris similarly insult the ocular drainage structures of both strains. Nevertheless, the B6 double-congenic mice were much less susceptible to IOP elevation than D2 mice. Probably because of their resistance to IOP elevation, the B6 double-congenics were not found to develop glaucoma. In fact, the degree of optic-nerve damage observed was largely similar to age-matched wild-type B6, indicating that the small degree of optic neuropathy observed was likely an independent phenomenon, such as the result of the normal aging process or a low-penetrance developmental abnormality. Experiments to understand this strain difference in IOP elevation are underway.

These experiments also suggest that the cytotoxicity mediated by *Tyrp1 *and *Gpnmb *does not kill the RGCs, and that iris disease and RGC death can be dissociated from each other. Therefore, RGC death in D2 mice appears to be a pressure-dependent process resembling the pathology of human glaucoma. In support of this, RGC death in this model is not uniformly distributed [48,49], as would be expected if damaged by an iris-derived diffusible toxin. Rather, it appears that RGCs are uniquely susceptible retinal neurons, and the pattern of damage is consistent with an insult to RGC axons in the optic nerve [17,49].

### Genetics and human PDS

The broader implications of the strain-specific phenotypes may be important for studies of the heredity and mechanism of human pigmentary glaucoma. In humans, PDS/PG exhibits strong hereditary associations [50-52]. A few families with clear inheritance of PDS are reported. However, the low incidence of PDS in family members of the majority of affected individuals suggests a more complex etiology, possibly influenced by multiple genetic and environmental factors. In some families, linkage was initially reported between the disease phenotype and markers on 7q35-q36 [50]. However, causative mutations at this locus have thus far not been found. Analysis of candidate genes suggested by experiments with D2 mice (such as *TYRP1 *and *GPNMB*) have also failed to identify any clear mutations in these families [53]. Thus, the disease in these families may be more complex than initially suspected. The lack of identified mutations in *GPNMB *and *TYRP1 *in these families does not preclude the involvement of these genes (or genes of similar function) in patients of other families or in the more common, more complex cases. It remains to be determined whether PDS/PG in other patient populations is caused by more tractable loci, or whether the identification of genes associated with human PDS/PG may simply necessitate approaches that can overcome genetic complexity.

### Genetic context and human glaucoma

From a mechanistic view, the dichotomy between the differing responses of the B6 double-congenics versus D2 mice is analogous to the differing responses of people with PDS. Many human eyes tolerate significant amounts of dispersed pigment without developing glaucoma [6,7]. Indeed, fewer than half of human eyes with pigment dispersion progress to pigmentary glaucoma [8-12]. This, along with the expected minor degree of aqueous humor outflow obstruction due to pigment itself [54,55], suggests that individual differences in the ocular reaction to dispersed pigment are important in determining whether pigment dispersion progresses to pigmentary glaucoma. The D2 and B6 congenic strains provide a tractable resource for mechanistically deciphering the ocular reactions that determine whether IOP becomes harmfully elevated after the drainage structures are insulted by dispersed pigment and iris debris. Future studies of these reactions will not only improve our mechanistic understanding of the disease, but may also suggest new classes of genes worthy of analysis among human PDS/PG patients.

### Experimental implications of strain specific effects

Our results highlight the profound importance of genetic background to complex diseases such as glaucoma. In addition to understanding human glaucoma, it is important to consider effects of genetic background when using animal models. As a genetic resource, these congenic mouse strains will help to address this issue. Until now, a strain-matched control for *Tyrp1 *– and *Gpnmb*-mediated disease has been problematic. For instance, although any standard inbred mouse strain that does not develop glaucoma can be utilized as a control for D2 mice, this experimental design does not distinguish whether any observed differences relate to the disease state of the eye, or are merely previously unappreciated strain-dependent background effects. Given the complexity of these glaucoma phenotypes, experiments comparing between different strain backgrounds or using segregating backgrounds can be misleading [13,56]. Importantly, the congenic mice generated here can be utilized with strain-matched normal B6 mice to perform appropriately controlled experiments.

### Pressure-independent RGC death in C57BL/6 mice

Pressure-independent RGC death has been reported in C57BL/6 mice and may depend on environment or substrain used. In wild-type C57BL/6 mice from the supplier Harlan (C57BL/6Hsd), an age-related reduction in the number of RGCs has been described [48]. By 18 months, the number of RGCs was reduced to approximately 46% that of young mice. In our current study, this age-related decline was absent in both the double-congenic B6.*Tyrp1*^*b *^*Gpnmb*^*R150X *^mice and normal B6 controls that all had a C57BL/6J background. Similarly, we did not detect glaucomatous RGC loss in another experiment where C57BL/6J mice were also aged to 19+ months [57]. This difference in RGC death between studies may be explained by potential environmental influences, differences in analysis protocols, or genetic differences in the substrain utilized.

## Conclusion

This work introduces B6 congenic strains that allow highly controlled experiments when studying glaucoma resulting from the *Tyrp1*^*b*^and *Gpnmb*^*R150X *^alleles. Using this resource, we uncovered a striking strain dependence of developing high IOP and glaucoma when challenged by a profound pigment-dispersing iris disease. In future experiments, it will be particularly important to identify the pathways and genes influencing IOP elevation in this setting.

## Methods

### Animal husbandry

Mice were obtained from The Jackson Laboratory (Bar Harbor, ME, USA). All animals were treated according to the guidelines of the Association for Research in Vision and Ophthalmology for use of animals in research. All experimental protocols were approved by the Animal Care and Use Committee of The Jackson Laboratory. Data for the congenic B6 strains are compared with those with a D2 background. Many of the data for the D2 mice (more than 1500 mice) have been previously reported but some new IOP data are included here. The congenic B6 mice were bred, aged and analyzed at the same time as over 300 D2 mice. The D2 mice were maintained on an NIH 31 diet with a fat content of 6%. To avoid obesity, mice with a B6 background had to be maintained on essentially the same NIH 31 diet but with a 4% fat content. Our studies have shown that D2 IOP and other glaucoma phenotypes do not differ with this small dietary difference (unpublished data). For all mice, the diet was provided ad libitum, and the water was acidified to pH 2.8–3.2. Mice were housed in cages containing white-pine bedding and covered with polyester filters. The environment was kept at 21°C with a 14-hour light:10-hour dark cycle.

### Generation of congenic strains and genotype analysis of congenic intervals

Chromosomal intervals of approximately 6–7 cM were selected for the regions flanking *Tyrp1 *(*D4Mit178 *to *D4Mit327*) and *Gpnmb *(*D6Mit74 *to *D6Mit355*). Mice were reiteratively bred to B6 mice and each successive generation genotyped to select breeders heterozygous for the respective D2 regions. This process was continued for 10 generations of backcrossing. At the 10th generation, mice were intercrossed and each region bred to homozygosity. Within the respective congenic intervals, the presence of the *Tyrp1*^*b *^allele was confirmed by assaying for a D2-derived polymorphism creating a *Taq*I restriction-enzyme site in exon 4 [35], and the presence of the *Gpnmb*^*R150X *^mutation was confirmed by assaying for a *Pvu*II restriction-enzyme site created by the D2-derived mutation [23]. The sizes of the congenic intervals were determined using genomic DNA prepared from homozygous mice at the 10th generation of backcrossing and assayed using polymorphic microsatellite markers with standard PCR conditions.

### Intraocular pressure measurement

IOP was measured using the microneedle method as previously described in detail [58,59]. All cohorts included male and female mice. Each mouse used in this study had only a single IOP measurement. Thus, each age group shown consists of cohorts of different individual mice. Because the IOPs of B6 mice are very consistent, B6 mice were interspersed with experimental mice during all experiments as a methodological control to ensure proper equipment calibration and performance. Statistical comparisons of IOP profiles generated from these strains utilize nonparametric data comparisons, with data grouped according to empirically set points relevant to these studies: 19 mmHg (i.e. > 2 SD above the mean for young pre-glaucomatous D2 mice) and 21 mmHg (a value commonly regarded as glaucoma suspect in humans) [41].

### Clinical and histologic analysis

Eyes were examined with a slit-lamp biomicroscope and photographed with a 40× objective lens. Phenotypic assessment of iris disease was determined by indices of iris atrophy and dispersed pigment following previously described criteria [18,22,23,60]. Transillumination defects were also measured (transillumination is an assay of iris disease whereby reflected light passing through depigmented areas of iris tissue are visualized as red light). However, because transillumination is influenced by multiple strain-specific traits not controlled in these studies (such as iris thickness), it was not the primary means of following disease progression across different genetic backgrounds.

Optic nerve cross sections were examined for glaucomatous damage using a modified *para*-phenylenediamine (PPD) staining protocol to stain the myelin sheath of all axons and the axoplasma of damaged axons [61]. Optic nerves were prepared for analysis by overnight fixation in phosphate-buffered glutaraldehyde/paraformaldehyde mixture at 4°C followed by overnight treatment in osmium tetroxide at 4°C. After osmification, nerves were rinsed twice for 10 minutes in 0.1 M phosphate buffer, once in 0.1 M sodium-acetate buffer, and dehydrated in graded ethanol concentrations. After embedding this tissue in resin, 1-μm-thick sections were stained in 1% PPD for 35–40 minutes.

Stained sections were compared using a previously described qualitative grading scale (including assessments of the approximate number of healthy axons, number of damaged axons, and scarring associated with gliosis) and used to perform quantitative axon counts [42,43]. The grading scale has previously been validated by comparisons to axon counts [42,43]. Quantitative axon counts imaged 18 non-overlapping fields at 1000× magnification, which were evenly spread throughout the cross-section of the nerve; the sum area of these fields was equal to 10% of the total nerve cross-sectional area. The majority of optic nerves were collected from mice within 48 hours of IOP measurement. Some mice with elevated IOPs were additionally age determined prior to assessment of the optic nerve. Each age group investigated contained samples from males and females, as well as left and right nerves.

### Genetic nomenclature and stocks

The official gene abbreviations, full gene names, alleles, and stocks utilized in this study include the following. The full name for the *Gpnmb *gene is glycoprotein (transmembrane) nmb. The *ipd *allele of *Gpnmb *results from the R150X premature stop codon mutation, *Gpnmb*^*R150X *^[23]. The full name for the *Tyrp1 *gene is tyrosinase-related protein 1. The *b *allele of *Tyrp1 *encodes two amino-acid substitutions, compared with the C57BL/6J-derived allele [35]. This D2 allele is also referred to as *isa *in the mouse genome database and elsewhere [22,23]. The DBA/2J stock utilized here was stock number 000671 from The Jackson Laboratory. The full name for the *Tyr *gene is tyrosinase. The *c-2J *mutation spontaneously arose within a C57BL/6J colony, resulting in a R77L amino acid substitution and also changing alternative splicing of the tyrosinase pre-mRNA [39,40]. The *Tyr*^*c*-2*J *^stock utilized here was C57BL/6J-*Tyr*^*c*-2*J*^/J (stock 000058 from The Jackson Laboratory).

## Authors' contributions

MGA carried out genetic crosses to generate the congenic and albino congenic strains, and performed the slit-lamp analysis of ocular phenotypes. MGA and SWMJ contributed to the conception, design, and coordination of all study components, analyzed the data, and wrote the manuscript. RTL analyzed IOP and optic nerve data and contributed to the design and presentation of the study. IMC measured IOP and participated in mouse breeding and genotyping. RSS, MM, and SWMJ participated in examining optic nerves. LAW managed the mouse colonies and coordinated experiments.
